# Dangerous for ferrets: lethal for humans?

**DOI:** 10.1186/1741-7007-10-10

**Published:** 2012-02-20

**Authors:** Peter C Doherty, Paul G Thomas

**Affiliations:** 1 Department of Microbiology and Immunology, University of Melbourne, Vic 3010, Australia; 2 Department of Immunology, St Jude Children's Research Hospital, Memphis, TN 38105, USA

## 

The influenza A viruses remain our most serious, known pandemic threat [1]. The possibility that a H5N1 high-pathogenicity avian influenza A virus (HPAI) could cross over to become established in humans has been a very real concern for more than a decade. As a consequence, it was big news at the recent Malta ESWI [2] meeting when Ron Fouchier from the Erasmus Medical Centre in Rotterdam announced that he had serially passaged an HPAI H5N1 virus in ferrets and achieved 'natural' ferret-to-ferret transmission. The first isolation ever (1933) of a human influenza A virus was in ferrets, and virologists generally regard these mustelids as the optimal model for most aspects of human influenza, including spread. It was not highlighted in Malta, but we learned later that Yoshi Kawaoka at the University of Wisconsin, Madison had similar findings, though he started with a virus that had been genetically modified in ways that might be expected to make it less virulent. It seems that others were also trying with reverse genetics approaches, but failed.

The Fouchier experiment was the talk of the Malta meeting, though not because of safety concerns. Influenza virologists had been debating for years whether these HPAI H5N1 viruses could ever change in a way that would allow them to transmit readily between human beings. Informed opinion was strongly divided. We all knew that, given exposure to what has been assumed to be a large virus dose from an infected bird, people can develop severe H5N1 disease, with a very high death rate (345 fatalities out of 584 cases since 2003) [3]. A few instances where family members may have been secondarily infected are on record, while two recent cases with no known history of avian contact have been reported from China. We are not there yet, but what these ferret adaptation studies suggest (though by no means prove) is that a 'human' H5N1 pandemic virus may indeed emerge from nature.

The issue of safety blew up much later when it came to publishing the Fouchier *et al*. findings. Should these genetic changes be 'out there' for all to see? Might that information be used by sophisticated bioterrorists? The furor about whether these ferret adaptation studies should ever have been done and, if so, whether the results should be openly published came as something of a surprise to us. Though some medical epidemiologists did raise the issue of risk, the resurrection of the catastrophic 1918 H1N1 virus by Jeff Taubengerger, Johan Hultin *et al*. more than a decade back met with general acclaim as an undoubted scientific achievement. The 1918 virus killed around 50 million people but nobody, so far as we recall, objected when (from 1999) segments of the virus sequence started to appear in the journals. Was the difference that there was, at that time, more trust in those who work in high security government laboratories? Then, much of the 1918 sequence was published before 9/11, 2001. The world has changed.

Of course, citizens, commentators, funding agencies and national governments have every right to insist that what is being done in laboratories constitutes no threat to the population at large. That has long been recognized, and organizations like the NIH, the FDA, universities and research institutes ensure that rigorous training requirements, review processes and safety checks/procedures are in place, along with the appropriate hardware and monitoring. Do such protocols need to be strengthened? That question is always appropriate, and should certainly be explored in the current climate.

One thing that has impressed us, as immunologists rather than virologists working with the influenza A viruses, is that while these pathogens transmit with such ease in nature, lab infections are rare [4-6]. Perhaps this reflects that the 'flu community is both very competent and operates under optimal conditions, but many other categories of viruses and bacteria are well-recognized threats for the (no doubt capable and well-equipped) microbiologists who work with them. Mistakes and accidents do happen and any institution that operates with highly pathogenic organisms must have clear protocols in place to deal with such situations. There is a suspicion that the 1977 'Russian pandemic' H1N1 virus may have spent the preceding 25-plus years in a laboratory deep-freeze [7]. Was this an escape from some 'hidden experiment', perhaps involving a live vaccine? The obvious inference is that it is essential for 'flu researchers everywhere to be open and interactive.

While we need to be assured that Taubenberger, Hultin, Fouchier, Kawaoka-type experiments are only done by responsible people working in safe, well-regulated institutions, the problem is that imposing highly restrictive constraints (BSL4 security, in space suits) means that few will be bothered to investigate these pathogens. As a consequence, we will know less about them. The reality is that if scientists want to be funded, they must be productive and publish. It is always easy for a talented researcher to say: 'too hard, too cumbersome, I'll just go on with something else.' Those reviewing the H5N1 situation might ask whether that has indeed happened with the resurrected 1918 virus.

This issue of appropriate security is what needs to be resolved, hopefully in the up-coming WHO discussion forum. Any agreement on if, when and where such experiments are to be done must obviously be at the global level. The era of 'western exceptionalism' in science is as dead as the dodo. The selective application of excessive requirements by, for example, Brussels and Washington will simply be an exercise in futility, and possibly dangerous.

What are the real risks? Is it realistic to think of using a virulent 'flu virus to create terror and social disruption? Influenza goes everywhere. How does a science terrorist who seeks to target a specific 'enemy' protect his own people? Are our security services on the watch for a rogue state that is vaccinating against H5N1 or some other dangerous pathogen? Quite frankly, as a tactical, strategic or bioterror weapon, our guess is that 'flu makes little sense. Still, the Fouchier mutations might be just what some mad molecular 'greenie' needs to go forward with his dastardly plan of depopulating the planet. Why delay though? Once Fouchier had talked about his experiments in Malta, all that our hypothetical maniac need do is to go out and buy a few dozen ferrets and some cages at a pet store, get an HPAI H5N1 virus from his source in an endemic country and do the experiment in a deserted farm or warehouse. Sounds like great TV. Anyway, virologists have known how to adapt 'flu viruses by serial passage or genetic reassortment for half a century, so what does Fouchier add?

What must be avoided at all costs is to initiate processes that limit the exchange of information in the influenza field. The overwhelming probability is that any 'human pandemic' H5N1 variant will come out of nature, not a laboratory. The combination of a superbly organized network, first class technology, well-established centers and dedicated professionals means that the global monitoring mechanisms for influenza are the best there can be. The Fouchier and Kawaoka studies identify mutations that these international 'flu detectives' will be watching for. The last thing the influenza surveillance community would want is for their work to become exclusive, especially if that leads to any reluctance to make newly isolated, dangerous H5N1 'field isolates' immediately available for general scrutiny.

## References

[B1] Osterholm MT Preparing for the next pandemic N Engl J Med 2005 352 1839 1842 10.1056/NEJMp058068 15872196

[B2] European Scientist Working Group on Influenza 2011 http://www.eswiconference.org/

[B3] WHO data as of 08 February 2012 http://www.who.int/influenza/human_animal_interface/EN_GIP_20120208CumulativeNumberH5N1cases.pdf

[B4] Sewell JL Laboratory infections and biosafety Clin Microbiol Rev 1995 8 389 405 7553572 10.1128/cmr.8.3.389 PMC174631

[B5] Singh K Laboratory-acquired infections Clin Infect Dis 2009 49 142 147 10.1086/599104 19480580 PMC7107998

[B6] Della-Porta A Laboratory accidents and breaches in biosafety - they do occur! Microbiol Aust 2008 29 62 65

[B7] Webster RG Bean WJ Gorman OT Chambers TM Kawaoka Y Evolution and ecology of influenza A viruses Microbiol Rev 1992 56 152 179 1579108 10.1128/mr.56.1.152-179.1992 PMC372859

